# Functional respiratory imaging, regional strain, and expiratory time constants at three levels of positive end expiratory pressure in an ex vivo pig model

**DOI:** 10.14814/phy2.13059

**Published:** 2016-12-06

**Authors:** William R. Henderson, Yannick Molgat‐Seon, Wim Vos, Rachel Lipson, Francisca Ferreira, Miranda Kirby, Cedric Van Holsbeke, Paolo B. Dominelli, Donald E. G. Griesdale, Mypinder Sekhon, Harvey O. Coxson, John Mayo, A. William Sheel

**Affiliations:** ^1^ Division of Critical Care MedicineVancouver General HospitalVancouverBritish ColumbiaCanada; ^2^ School of KinesiologyVancouverCanada; ^3^ FluidDALeuvenBelgium; ^4^ Emmes CanadaVancouverCanada; ^5^ RadiologyThe University of British ColumbiaVancouverBritish ColumbiaCanada; ^6^ School of KinesiologyUniversity of British ColumbiaVancouverBritish ColumbiaCanada; ^7^ Division of Critical Care MedicineVancouver General HospitalVancouverBritish ColumbiaCanada; ^8^ Centre for Heart Lung InnovationSt Paul's HospitalUniversity of British ColumbiaVancouverBritish ColumbiaCanada; ^9^ Department of RadiologyVancouver General HospitalUniversity of British ColumbiaVancouverBritish ColumbiaCanada

**Keywords:** Positive end‐expiratory pressure, strain, time constant

## Abstract

Heterogeneity in regional end expiratory lung volume (EELV) may lead to variations in regional strain (*ε*). High *ε* levels have been associated with ventilator‐associated lung injury (VALI). While both whole lung and regional EELV may be affected by changes in positive end‐expiratory pressure (PEEP), regional variations are not revealed by conventional respiratory system measurements. Differential rates of deflation of adjacent lung units due to regional variation in expiratory time constants (*τ*_E_) may create localized regions of *ε* that are significantly greater than implied by whole lung measures. We used functional respiratory imaging (FRI) in an ex vivo porcine lung model to: (i) demonstrate that computed tomography (CT)‐based imaging studies can be used to assess global and regional values of *ε* and *τ*_E_ and, (ii) demonstrate that the manipulation of PEEP will cause measurable changes in total and regional *ε* and *τ*_E_ values. Our study provides three insights into lung mechanics. First, image‐based measurements reveal egional variation that cannot be detected by traditional methods such as spirometry. Second, the manipulation of PEEP causes global and regional changes in R, E, *ε* and *τ*_E_ values. Finally, regional *ε* and *τ*_E_ were correlated in several lobes, suggesting the possibility that regional *τ*_E_ could be used as a surrogate marker for regional *ε*.

## Introduction

Mechanical ventilation is used in the care of surgical and medically ill patients. Current best practice assesses management of mechanical ventilation based on indices of oxygenation or on aggregate measures of pulmonary function such as pressure–volume curves or transpulmonary pressure (ARDSNetwork 2000; Brower et al. 2004; Guérin 2013; Goligher et al. 2014). While pragmatic, these strategies do not account for regional variation in lung tissue parameters such as strain (*ε*), a crucial determinant of local lung injury (Eissa et al. 1991; Schiller et al. 2001; Hickling 2002; Otto et al. 2008; Mertens et al. 2009; Loring et al. 2010; Kaczka et al. 2011a *,* b). Strain (*ε*) is the ratio of tidal volume (*V*_T_) to end‐expiratory lung volume (EELV). High *ε* levels have been associated with ventilator‐associated lung injury (VALI) (Protti et al. 2011; González‐López et al. 2012). The heterogeneity in regional EELV due to atelectasis may lead to variations in regional *ε* (Cressoni et al. 2014). While both whole lung and regional EELV may be affected by changes in positive end‐expiratory pressure (PEEP), regional variations are not revealed by conventional respiratory system measurements.

The ratio of the resistance (R) to elastance (E) of a lung unit during passive deflation is, defined as the expiratory time constant (*τ*_E_). Differential rates of deflation of adjacent lung units due to regional variation in *τ*_E_ may create localized regions of *ε* that are significantly greater than implied by whole lung measures of pressure or *ε* (Mead et al. 1970; Kaczka et al. 2011a; Perchiazzi et al. 2011; Cressoni et al. 2014).

In recent studies, functional respiratory imaging (FRI) information has been obtained by combining anatomic computed tomography (CT) images with functional information calculated using computational fluid dynamics (CFD). To achieve this, numerical flow equations (Navier‐Stokes equations) are solved in subject‐specific computational grids, based on segmented three‐dimensional models of the airways and lungs, using subject‐specific boundary conditions (Lin et al. 2009; De Backer et al. 2008). The result of FRI is a local description of volume, pressure and flow characteristics throughout the entire respiratory system, which has proven to be more sensitive than conventional lung function measures (De Backer et al. 2012; Vos et al. 2013). Furthermore, FRI provides novel insights in the mode of action of new compounds that have effects that are hard to analyze via traditional lung function testing (De Backer et al. 2014; Vos et al. 2016). Thus, FRI provides a method by which local *ε* and *τ*_E_ values may be assessed.

Our understanding of the complexities of how PEEP causes regional changes in strain and tau is incomplete and advances have been methodologically limited. As such, the purpose of this study was to use a novel imaging method to assess regional values of *ε* and *τ*_E_ and test the hypothesis that changes in PEEP would have distinct effects on a per lobe basis. To this end, we used FRI in an ex‐vivo porcine lung model to: (i) demonstrate that CT‐based imaging studies can be used to assess global and regional values of *ε* and *τ*_E_ and, (ii) demonstrate that the manipulation of PEEP will cause measurable changes in total and regional *ε* and *τ*_E_ values.

## Methods

### Ethical approval

All experiments were approved by the Animal Research Committee of the University of British Columbia, Vancouver, British Columbia and conformed guidelines outlined by the Physiological Society (Grundy 2015).

### Ex‐vivo lung preparation

After participation in a surgical skills training session, euthanasia with pentobarbital sodium (120 mg kg^−1^ intravenous) was achieved in five adult female Yorkshire X pigs. Death was confirmed by the absence of a pulse and cardiac electrical activity on continuous surface electrocardiography. The lungs and trachea of were removed *en block* through a sternal incision, and an endotracheal tube (9.0 mm internal diameter) was placed into the trachea. The lungs and trachea were then suspended from a nonmetallic scaffold inside of a CT scanner (Aquilion One Volumetric CT scanner; Toshiba Medical Systems, Tustin, CA). The lungs were then initially ventilated (Puritan‐Bennett 7200; Covidien, Dublin, Ireland) with 0 cm H_2_O of PEEP, using a fraction of inspired oxygen of 0.21, *V*_T_ of 6 mL kg^−1^ of body weight, and a breathing frequency (*f*_b_) of 12 breaths min^−1^. Inspiratory and expiratory flows (${\dot{V}}_{I}$ and ${\dot{V}}_{E}$) were measured using a heated pneumotachograph (Model 3813; Hans Rudolph, Shawnee, KS) placed between the ventilator tubing wye and the proximal end of the endotracheal tube. Inspiratory flows were held constant at 45 L min^−1^ with a square waveform. The pneumontachometer was calibrated with a 1 L calibration syringe prior to each trial. Inspiratory and expiratory volumes (*V*_I_ and *V*_E_) were obtained by numerical integration of the flow signals.

### Intervention

The lungs were ventilated as described above with PEEP levels of 0, 5 and 10 cm H_2_O. The order of PEEP levels was randomly assigned, and 10 min of ventilation at each PEEP level occurred prior to recording images and physiological data.

### Image acquisition and processing

**Figure 1:**
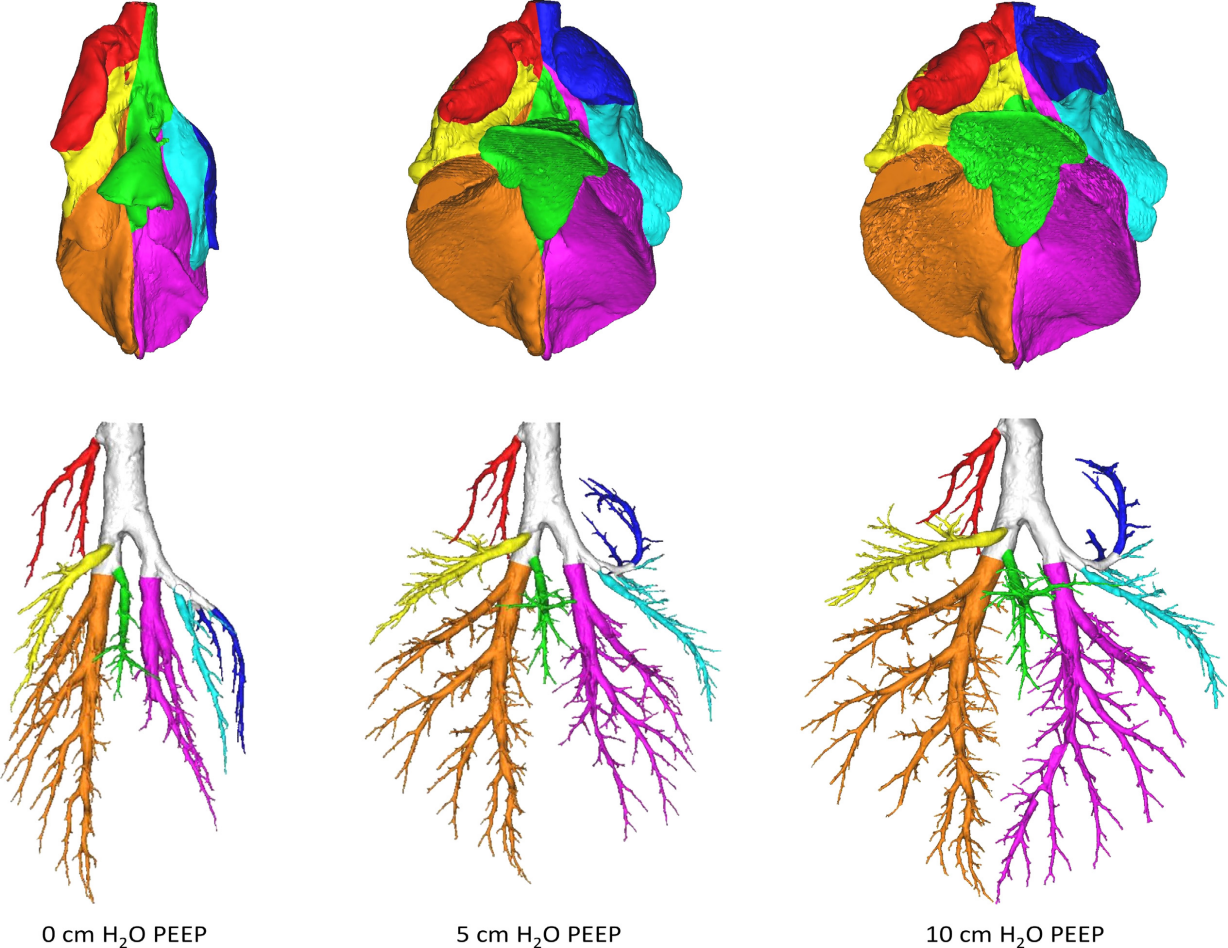
Pictoral representation of lung lobes and airways using composite 3D airway images at three positive end expiratory pressures. PEEP 0/5/10, positive end expiratory pressure of 0/5/10 cm H_2_O. Red, right anterior lobe; Yellow, right caudal lobe; Orange, right diaphragmatic lobe; Green, right internal lobe; Purple, left diaphragmatic lobe; Light blue, left caudal lobe; Dark blue, left anterior lobe.

All lungs underwent CT imaging at multiple time points during inflation and deflation on the mechanical ventilator. Imaging was performed with the lung suspended in the upright position and was gated to begin at the beginning of inflation and end at the cessation of inflation. The CT settings were as follows: tube voltage, 120 kV; tube current, 200 mAs; rotation time, 0.35 sec; field of view, 400.4 mm; slice thickness, 0.50 mm; pixel spacing 0.782 mm; and convolution kernel, FC51. The images were acquired without moving the table (pitch: 0) and the acquisition began immediately prior to lung inflation and concluded after complete cessation of gas flow following passive deflation. Imaging data was converted into 3D models of airways and lung lobes using Mimics 15 (Materialise, Leuven, Belgium) a previously validated software package (Food and Drug Administration, K073468; Conformité Européenne certificate, BE 05/1191.CE.01) (Video 1). Airways were segmented using directional thresholding with automated leakage detection. Lungs were split into lobes by identification of the fissure lines from the CT scan. For both airway and lung lobe segmentation, manual updating of the automated algorithms was performed when needed. Lungs lobes and the respiratory tract could be extracted at several time points during inflation and deflation (Fig. 1). For data analysis, we used the end‐expiratory scan for both lobar and airway analysis and the end‐inspiratory scan for lobar analysis only.

We identified lobes as follows: right and left anterior lobes (RAL and LAL), right and left caudal lobes (RCL and LCL), right and left diaphragmatic lobes (LDL and LDL), and the right internal lobe (RIL) (Fig. 1). Airways were trimmed perpendicular to the local centerline in order to prepare the model for CFD airflow analysis. Airway models were split into a central part and airways leading to each previously defined lobe (Fig. 1).

### Calculation of strain and time constant values using CT data

Using the derived 3D lobar models, we calculated the volume of each lobe at end‐inspiratory lung volume (EILV) and EELV. Regional *ε* for each lobe was calculated as follows: (EILV − EELV)/EELV using the appropriate lobar volumes. For example, the *ε* of the RDL (*ε*_RDL_) was calculated as (EILV_RDL_ − EELV_RDL_)/EELV_RDL._ Total respiratory system EELV (EELV_RS_) and EILV (EILV_RS_) were calculated by determining the sum of all lobar volumes. Total respiratory system *ε* (*ε*_RS_) was calculated as follows: (EILV_RS_ − EELV_RS_)/EELV_RS_.

Expiratory time constants were calculated by determining the quotient of R and E. Resistance was defined as the total pressure drop needed to drive flow through an airway section. Regional airflow was obtained from the total airflow ((EILV_RS_ − EELV_RS_)/time of deflation) combined with the internal airflow distribution calculated on a lobar basis by: (EILV_RDL_ − EELV_RDL_)/(EILV_RS_ − EELV_RS_). Expiratory laminar steady CFD calculations were carried out using velocity inlets (inlet velocity = flow rate through region/total area of inlet in the region) at the terminal bronchi and a pressure outlet (total pressure = PEEP) at the trachea. From the CFD calculation, the pressure drop over each specific lobar region was obtained. Elastance was defined as the pressure change needed to obtain a known volume. The volume change was given by EILV‐EELV and the pressure change was the pressure drop in the trachea throughout deflation. CFD simulations were performed in Fluent 14.0 (Ansys Inc, Canonsburg, PA).

### Statistical analysis

For lobe‐specific models, lobe, PEEP and their interaction were included as main effects with a random effect for pig included to account for the possibility of intra‐animal correlation. Testing for significance of fixed effects was done with *F*‐Tests and type III sums of squares. Total lung measurements were not included in lobe‐specific models and were modeled separately, using a similar approach. All testing of differences in least squared means was adjusted for multiple comparisons, using the Tukey–Kramer method. *P*‐values less than 0.05 were considered significant.

Separate mixed effect models to assess the relationship between tau and strain were fit for each lobe and for the total lung so that separate *R* ^2^ values could be obtained. Each model included *τ*_E_ as the response, *ε* as the predictor, and a random effect for pig. The pseudo‐*R* ^2^ value $R_{\mathrm{LMM}(m)}^{2},$ proposed by Nakagawa and Schielzeth for mixed effects models (Nakagawa and Schielzeth 2013), is reported for each lobe and for the entire lung in Figure Y, where $R_{\mathrm{LMM}(m)}^{2}$ measures the proportion of the variance of *τ*_E_ explained by *ε* based on the fitted mixed effect model. $R_{\mathrm{LMM}(m)}^{2}$ values of 1 indicate perfect correlation between tau and strain, while a value of 0 indicates no correlation between them. Testing the significance of the relationship between tau and strain was performed with *F*‐tests and type III sums of squares, and the Benjamini–Hochberg significance level was used to determine if the relationship between *τ*_E_ and *ε* was statistically significant while adjusting for multiple comparisons.

## Results

### Global resistance, elastance, strain and time constants

When considering the whole respiratory system using FRI, R, E, *ε* and *τ*_E_ were all affected by PEEP (*P* < 0.05 for each). After adjusting for multiple comparisons, R at PEEP 0 was significantly higher than at PEEP 10 (*P* < 0.05). Elastance was significantly higher at PEEP 10 compared to PEEP 0 and 5 (*P* < 0.01), *ε* was higher at PEEP 0 than at PEEP 5 or 10 (*P* < 0.05), and *τ*_E_ at PEEP 0 and 5 was significantly higher than at PEEP 10 (*P* < 0.05).

### Regional resistance, elastance, strain and time constants

**Figure 2:**
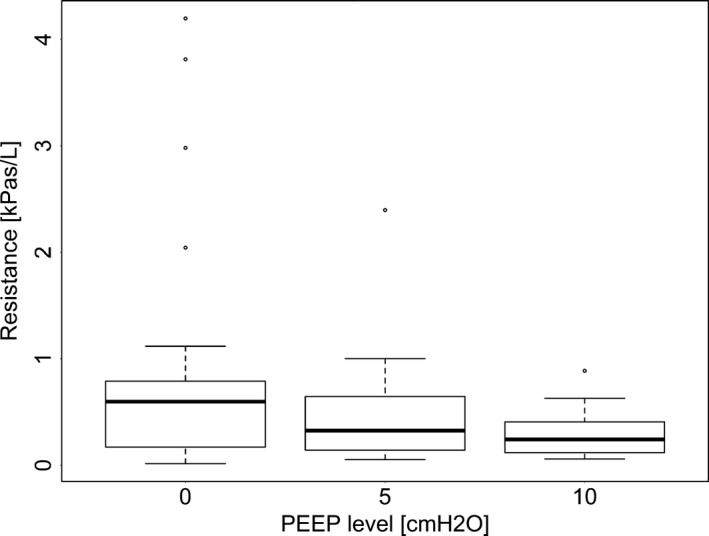
The effect of positive end expiratory pressure (PEEP) in expiratory resistance as calculated by functional respiratory imaging (FRI). The extremes of the box represent the quartiles and the black line represents the median. The whiskers extend to the most extreme data point which is no more than 1.5 times the interquartile range from the box. Open circles represent data points outside of this range**.**

**Figure 3:**
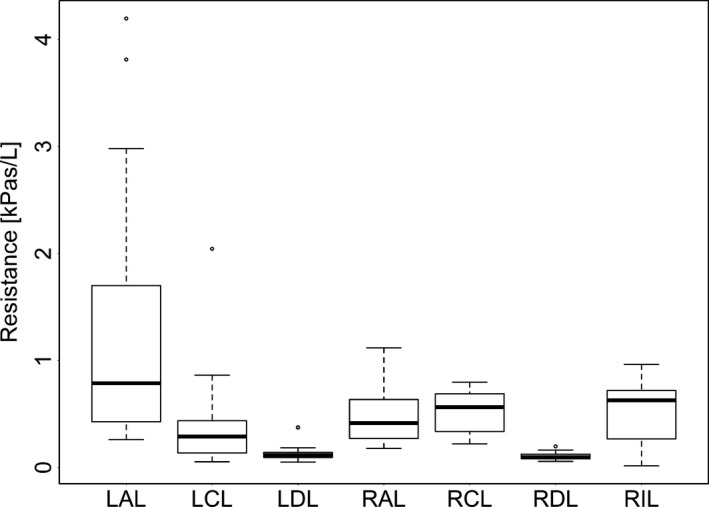
The effect of lobe on expiratory resistance as calculated by functional respiratory imaging (FRI). RAL, right anterior lobe; RCL, right caudal lobe; RDL, right diaphragmatic lobe; RIL, right internal lobe; LDL, left diaphragmatic lobe; LCL, left caudal lobe; LAL, left anterior lobe. The extremes of the box represent the quartiles and the black line represents the median. The whiskers extend to the most extreme data point which is no more than 1.5 times the interquartile range from the box. Open circles represent data points outside of this range.

In the model including lobe, R was affected by PEEP, lobe, and the interaction of PEEP and lobe (*P* < 0.0001); higher PEEP resulted in reduced resistance, but the effect of PEEP on R differed between lobes. Data regarding R values per PEEP setting and per lobe are in Figures 2 and 3, respectively. Specifically, R in LAL was significantly different from all other lobes (*P* < 0.0001) and R at PEEP 0 was significantly higher than PEEP 5 and 10 (*P* < 0.01).

**Figure 4:**
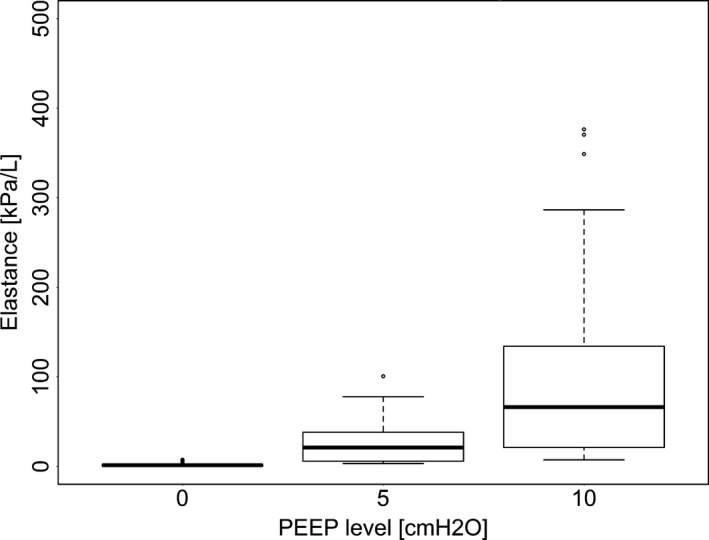
The effect of positive end expiratory pressure (PEEP) in lung elastance as calculated by functional respiratory imaging (FRI). The extremes of the box represent the quartiles and the black line represents the median. The whiskers extend to the most extreme data point which is no more than 1.5 times the interquartile range from the box. Open circles represent data points outside of this range.

**Figure 5:**
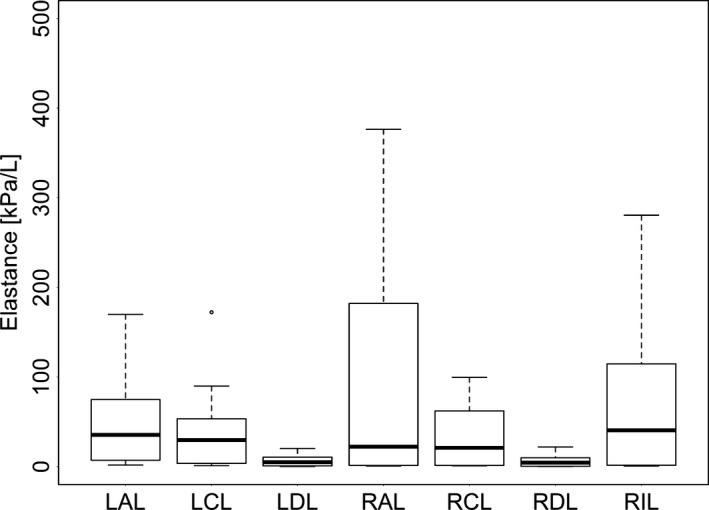
The effect of lobe in lung elastance as calculated by functional respiratory imaging (FRI). RAL, right anterior lobe; RCL, right caudal lobe; RDL, right diaphragmatic lobe; RIL, right internal lobe; LDL, left diaphragmatic lobe; LCL, left caudal lobe; LAL, left anterior lobe. The extremes of the box represent the quartiles and the black line represents the median. The whiskers extend to the most extreme data point which is no more than 1.5 times the interquartile range from the box. Open circles represent data points outside of this range.

Elastance was different for different levels of PEEP (Fig. 4, *P* < 0.01) and lobe (Fig. 5, *P* < 0.0001), but the interaction was not significant (*P* = 0.18); the relationship between PEEP and lobe was similar for all lobes. However, some specific differences did exist: E in RAL was significantly higher than in LAL, LCL, LDL, RCL, and RDL (*P* < 0.01) and was greater in RIL than in LDL and RDL (*P* < 0.01). In addition, E was significantly higher at PEEP 10 than at PEEP 0 or 5 (*P* < 0.05).

**Figure 6:**
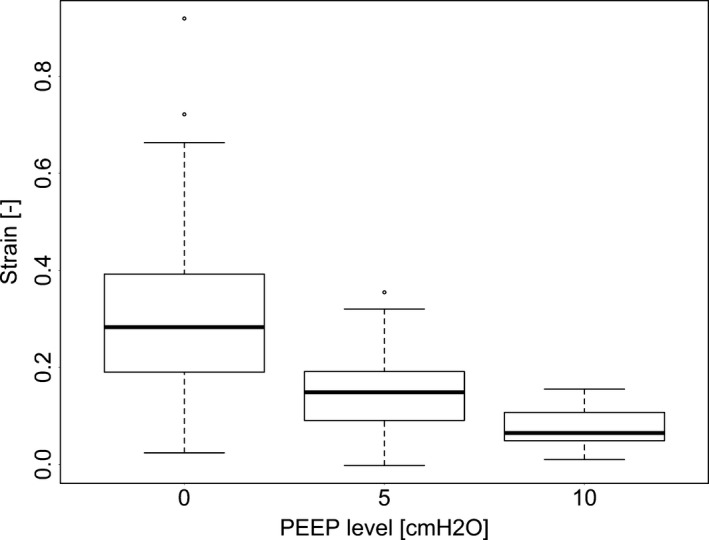
The effect of positive end expiratory pressure (PEEP) on strain as calculated by functional respiratory imaging (FRI). The extremes of the box represent the quartiles and the black line represents the median. The whiskers extend to the most extreme data point which is no more than 1.5 times the interquartile range from the box. Open circles represent data points outside of this range.

**Figure 7:**
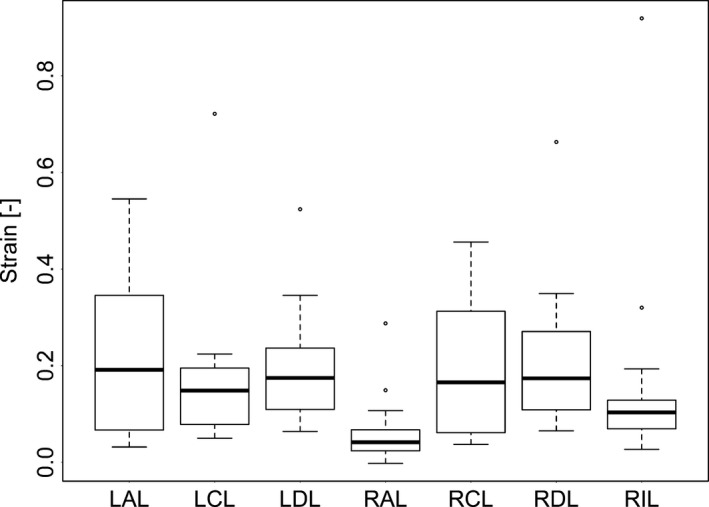
The effect of lobe on strain as calculated by functional respiratory imaging (FRI). RAL, right anterior lobe; RCL, right caudal lobe; RDL, right diaphragmatic lobe; RIL, right internal lobe; LDL, left diaphragmatic lobe; LCL, left caudal lobe; LAL, left anterior lobe. The extremes of the box represent the quartiles and the black line represents the median. The whiskers extend to the most extreme data point which is no more than 1.5 times the interquartile range from the box. Open circles represent data points outside of this range.

Strain was affected by PEEP (Fig. 6, *P* < 0.05) and lobe (Fig. 7, *P* < 0.0001) but not by the interaction of PEEP and lobe (*P* = 0.57). RAL was found to be significantly different from LAL and RDL with respect to *ε* (*P* < 0.05). Strain was highest at PEEP 0 compared to 5 and 10 (*P* < 0.0001) and was also higher at PEEP 5 than at PEEP 10 (*P* < 0.05).

**Figure 8:**
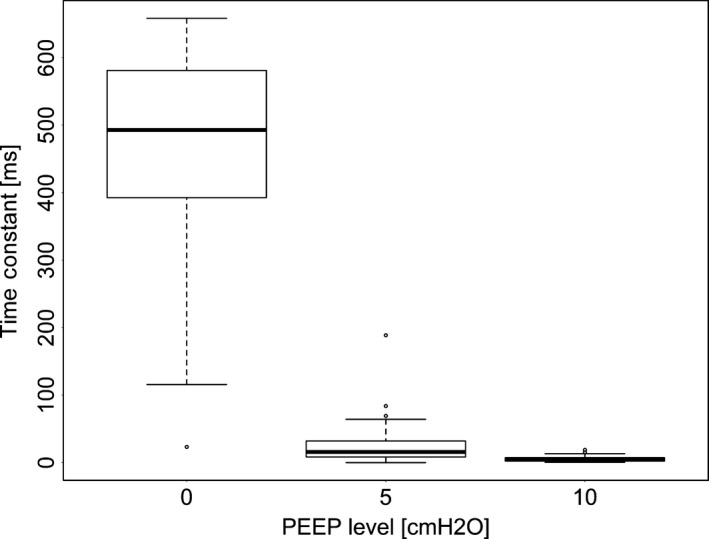
The effect of positive end expiratory pressure (PEEP) on the expiratory time constant as calculated by functional respiratory imaging (FRI). The extremes of the box represent the quartiles and the black line represents the median. The whiskers extend to the most extreme data point which is no more than 1.5 times the interquartile range from the box. Open circles represent data points outside of this range.

**Figure 9:**
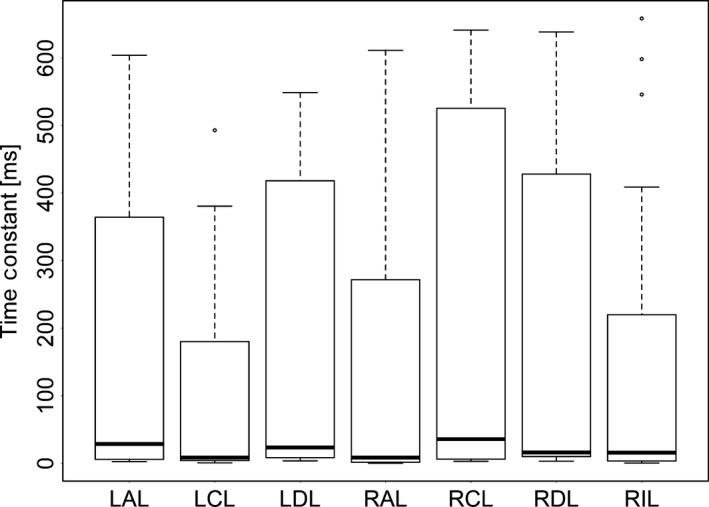
The effect of lobe on the expiratory time constant as calculated by functional respiratory imaging (FRI). RAL, right anterior lobe; RCL, right caudal lobe; RDL, right diaphragmatic lobe; RIL, right internal lobe; LDL, left diaphragmatic lobe; LCL, left caudal lobe; LAL, left anterior lobe. The extremes of the box represent the quartiles and the black line represents the median. The whiskers extend to the most extreme data point which is no more than 1.5 times the interquartile range from the box. Open circles represent data points outside of this range.

Consistent with the relationship of *τ*_E_ to R, *τ*_E_ values were affected by PEEP (Fig. 8, *P* < 0.01), lobe (Fig. 9, *P* < 0.01) and the interaction between PEEP and lobe (*P* < 0.05). The values of *τ*_E_ in LAL was also significantly higher than in all other lobes (*P* < 0.05) and *τ*_E_ was higher at PEEP 0 than at PEEP 5 or 10 (*P* < 0.05).

### Relationship between strain and time constants

After adjusting for multiple comparisons, there was evidence of a relationship between *τ*_E_ and *ε* in lobes LAL, LCL, RAL, RCL, and RIL (*P* < 0.01, Benjamini–Hochberg significance level 0.03). The pseudo‐*R* ^2^ between *τ*_E_ and *ε* was high in lobes LCL and RAL ($R_{\mathrm{LMM}(m)}^{2}$ > 0.9), moderate in lobes LAL, RCL, and RIL (0.4 < $R_{\mathrm{LMM}(m)}^{2}$ > 0.9) and low in lobes LDL, RDL, and in the total lung measurement ($R_{\mathrm{LMM}(m)}^{2}$ < 0.2).

## Discussion

Our study used FRI to quantify differences between global and regional values of R, E, *ε* and *τ*_E_ and provides three insights into lung mechanics. First, image‐based measurements reveal regional variation that cannot be detected by traditional methods such as spirometry. Second, the manipulation of PEEP causes global and regional changes in R, E, *ε* and *τ*_E_ values. Finally, regional *ε* and *τ*_E_ were correlated in several lobes, suggesting the possibility that regional *τ*_E_ could be used as a surrogate marker for regional *ε*. In sum, our experimental findings provide insight into the physiological complexities of mechanical ventilation where we found that PEEP causes significant regional differences in resistance, strain and expiratory time constants that are not detectable with conventional methods.

### Global and regional strain with FRI

Recognition of the deleterious effects of large *V*_T_ during mechanical ventilation has increased over the last twenty years, with recommendations to minimize *V*_T_ in an attempt to minimize *ε*_RS_ (ARDSNetwork 2000). Recent studies (Chiumello et al. 2008; Protti et al. 2011) have shown that *ε*_RS_ is a primary driver of VALI, and that *V*_T_ is a poor surrogate for *ε*_RS_ (Gattinoni et al. 2003; Santana et al. 2009; González‐López et al. 2012). Therefore, to mitigate VALI, investigators have attempted to more accurately measure important mechanical parameters of the lung such as *ε*. Our results demonstrate that *ε*_RS_ decreased with increasing PEEP, an observation that has been previously documented and which was due to increased EELV_RS_ in our experiment (Blankman et al. 2016). More important than the fact that the techniques described in our study allow the measurement of *ε*_RS_, is the ability to observe regional *ε*. Heterogeneity in regional *ε* has previously been observed using methods such as CT scans, electrical impedance plethysmography and forced oscillation techniques (Kaczka et al. 2011b; Pulletz et al. 2012; Wolf et al. 2013; Cressoni et al. 2014; da Paula et al. 2016). We found that varied widely, with the highest strain lobes exposed to approximately threefold the strain of the lowest strain lobes (Fig. 7). Both PEEP and lobe independently affected the *ε* measured in individual lobes. The clinical implications of interlobe variability in *ε* may be significant. For example, clinicians may use whole respiratory system measurements (such as *ε*_RS_) in an attempt to minimize new lung injury during mechanical ventilation, but may unwittingly still subject some lung regions to high *ε* levels_._

### Global and regional time constants with FRI

During passive deflation, *τ*_E_ conveys information about pulmonary mechanics that may guide therapy (Lourens et al. 2000; Al‐Rawas et al. 2013). We calculated expiratory values *τ*_E_ using this novel application of FRI. Deflation was more rapid with increases in PEEP (Fig. 8) and these decreases in *τ*_ERS_ were due to decreases in R, rather than increases in E (Figs. 2 and 4). Historically, methods of measuring *τ*_E_ were limited to whole lung measures based upon the assumption that the lung is isotropic, and are therefore unable to address regional differences in lung mechanics. Kaczka et al. (2005) have demonstrated that regional values of R and E (and by implication, regional values of *τ*_E_) may vary with both f_b_ and pressure. Similarly, our data demonstrate that there is substantial variation in *τ*_E_ between lobes, and that this variation is not well represented by the global measure (*τ*_ERS_). The significant interaction terms seen in our statistical model indicate that both PEEP and lobe affected *τ*_E_ in the per lobe analysis. The clinical implication of these findings is that some lobes may require significantly longer to fill or empty than *τ*_ERS_ would suggest, potentially leading to areas of shunt or deadspace. This raises that possibility that a clearer understanding of the regional variation in *τ*_E_ could guide clinicians in modifying the parameters of mechanical ventilation used in patients with significant regional variation in *τ*_E._

### Correlation between strain and time constants

Across all PEEP levels, the correlation between *τ*_E._and *ε* was very strong in lobes RAL and LCL, short *τ*_E._correlating with small *ε*. This relationship was weak for the total lung and reflects that the heterogeneous nature of the relationship between *τ*_E_ and *ε* in the different lobes. This limited finding provides some support for the idea that regional *τ*_E_ variation could be a useful surrogate for regional *ε* variation. However, this possibility requires significant clarification in future studies before it has clinical utility.

### Limitations

This study demonstrates the utility of FRI to measure regional heterogeneity in both *ε* and *τ*_E_, but some potential limitations should be acknowledged. First, the best manner in which to calculate *ε* in studies of mechanical ventilation that utilize PEEP is controversial. During normal breathing without PEEP, *ε* is the ratio of *V*_T_ to functional residual capacity (FRC). The application of PEEP will increase the resting lung volume by some additional volume (*V*_PEEP_). Several investigators have added *V*_PEEP_ to the distending volume and therefore calculate *ε* as (*V*_T_+*V*_PEEP_)/FRC (Protti et al. 2009; Gattinoni et al. 2012). Underlying this method is the rationale that *V*_PEEP_ distends the lung beyond FRC and thereby causing deformation. Others have calculated *ε* as *V*_T_/EELV, where EELV includes both FRC and V_PEEP_ (Mentzelopoulos et al. 2005; Brunner and Wysocki 2009; da Paula et al. 2016). The latter approach was used in this study for two reasons. First, stress relaxation may allow the lungs to achieve a new resting volume (that is without *ε* at end‐expiration at different PEEP levels) (Fuld et al. 2008). Recent analyses that indicate that static deformation due to *ε* (due to PEEP) may be less injurious than dynamic volume changes due to *ε* (from *V*_T_) support this view (Protti et al. 2013). Moreover, the principal utility of strain calculations is to mitigate new lung injury, particularly in patients with significant lung injury or acute respiratory distress syndrome. It is difficult to measure EELV in these PEEP‐dependent patients due to the risk of hypoxemia when PEEP is removed. This study thus replicates the clinical reality in the treatment of such patients. Finally, this study occurred in cadaveric lungs that were not contained within a chest wall. In this situation both EELV and E_RS_ will be lower than in intact subjects. These factors will increase *ε* measurements (particularly in the zero PEEP condition), lengthen *τ*_E_ values, and alter the distribution of lobar strains as compared to live animals. Second, in the current experimental set up, the lungs were suspended vertically as opposed to horizontally. This may have altered the interlobar *ε* distribution as compared to intact subjects. Finally, at end‐expiration with zero PEEP, alveolar ducts and small conducting airways may be subjected to cyclic derecruitment and recruitment. This could expose them to nonlinear dynamics that would not be captured in our computational model.

## Conclusion

Mechanical ventilation may precipitate VALI through the creation of high global *ε*. Both theory and experimental data suggest that whole lung measures of strain do not accurately represent regional pulmonary mechanics. Our data show that FRI can demonstrate the significant differences between regional and global measures of *ε* and *τ*_E._ We found limited evidence that *ε* and *τ*_E_ are correlated. While the clinical importance of these data may be significant, further studies are required to clarify their use in clinical practice.

## Conflict of Interest

All authors affirm that they have no competing interests with respect to the subject matter of this manuscript. WV is shareholder of FLUIDDA NV. CVH and FF are employed by FLUIDDA NV.
